# Metabolic risk factor profile associated with use of second generation antipsychotics: a cross sectional study in a community mental health centre

**DOI:** 10.1186/1471-244X-6-11

**Published:** 2006-03-16

**Authors:** Ilaria Tarricone, Michela Casoria, Beatrice Ferrari Gozzi, Daniela Grieco, Marco Menchetti, Alessandro Serretti, Manjola Ujkaj, Francesca Pastorelli, Domenico Berardi

**Affiliations:** 1 Institute of Psychiatry, Bologna University, Viale C. Pepoli 5, 40123 Bologna, Italy

## Abstract

**Background:**

Second generation antipsychotics (SGA) have demonstrated several advantages over first generation antipsychotics (FGA) in terms of positive, negative, cognitive, and affective symptoms and a lower propensity for extrapyramidal side effects. Despite these undeniable advantages, SGA have been associated with causing and exacerbating metabolic disorders, such as obesity, diabetes, and hyperlipidemia. This cross sectional study aimed to evaluate the metabolic risk factor profile associated with use of SGAs in comparison with non -treated control patients.

**Methods:**

The study was carried out at a Community Mental Health Centre (CMHC) in Bologna. The study subjects were outpatients with serious mental disorders treated with SGA (clozapine, olanzapine, risperidone, quetiapine). A sample of adult men and women suffering from idiopathic hyperhydrosis, without psychiatric history or antipsychotic treatment, were randomly selected from outpatients of the Department of Neurology in Bologna as a reference group. We investigated differences among the treatment and reference groups for glycaemia, cholesterolaemia and triglyceridaemia levels.

**Results:**

The study sample was composed of 76 patients, 38 males and 38 females. The reference group was composed of 36 subjects, 19 females and 17 males. All patients treated with SGAs had higher mean glycaemia and triglyceridaemia and a significantly higher risk of receiving a diagnosis of hyperglycaemia and hypertriglyceridaemia than the reference group. We did not find any differences in mean glycaemia or mean triglyceridaemia levels among treatment groups. Patients with clozapine had a significantly higher mean BMI value and rate of obesity than patients treated with other SGAs.

**Conclusion:**

The rate of obesity and metabolic disorders observed in this study were higher than the prevalence in the control group and similar to that previously reported in psychiatric samples; these findings imply *per se *that more attention should be paid to the metabolic condition of psychiatric patients. In line with the International Consensus Conferences we recommend that monitoring of weight, fasting plasma glucose, cholesterol and triglyceride levels be obtained in routine clinical practice with all antipsychotics.

## Background

In the last decade, second generation antipsychotics (SGA) have shown several advantages over first generation antipsychotics (FGA) in terms of positive, negative, cognitive, and affective symptoms and a lower propensity for extrapyramidal side effects [1,2]. Despite these undeniable advantages, SGA have been associated with causing and exacerbating metabolic disorders, such as weight gain/obesity, diabetes, and hyperlipidaemia [3-6]. Hyperlipidaemic effects include both hypertriglyceridaemia and hypercholesterolaemia, although relatively greater effects may be seen in triglyceridaemia [5,6]. Overweight, hyperglycaemia and hyperlipidaemia are risk factors for cardiovascular events, such as myocardial infarction and stroke, and are especially relevant in patients with schizophrenia because they tend to exhibit other major risk factors such as smoking and a sedentary lifestyle [4,7]. Moreover, it has been reported that weight gain is one of main side effects that will cause patients to discontinue treatments, thus exposing patients with schizophrenia to an increased risk of relapse [3,7].

Since most SGA have similar efficacy, side effect profiles become crucial when choosing treatment. According to a recent US consensus statement [3], the SGA vary in their propensity to cause obesity, hyperglycaemia and hyperlipidaemia, with clozapine and olanzapine showing the greatest effects, risperidone and quetiapine having intermediate effects, and aripiprazole and ziprasidone having lower effects. On the other hand, European [8] and Australian [9] consensus statements did not recognize any difference among SGA as to their propensity to cause metabolic side effects. Moreover, the American consensus statement itsel acknowledges the caveat that aripiprazole and ziprasidone have fewer long-term data due to the limited amount of time they have been on the market [3].

The present cross sectional study aims to evaluate the metabolic risk factor profile of outpatients with mental disorders treated with SGA in the everyday clinical practice of a Community Mental Health Centre (CMHC) in Bologna, and to compare this study population with a reference group of un-treated non-psychiatric outpatients.

## Method

This was an open-label, cross-sectional study carried out from January 2005 to May 2005 at a Community Mental Health Centre (CMHC) in Bologna with a catchment area of about 60,000 people. Subjects for the present study were outpatients with serious mental disorders on maintenance treatment with the SGA available in Italy in 2005 (clozapine, olanzapine, risperidone, quetiapine). Since no formal exclusion criteria were applied, all patients treated with SGA were eligible for the study. Informed consent was obtained from the patients. This study was performed with the approval of the Bologna University ethics committee, in compliance with the Helsinki Declaration.

### Assessment

Clinical records were used for psychiatric diagnoses according to DSM IV [10] criteria. Patients' socio-demographic, clinical and treatment information (current antipsychotic, doses and duration of treatment; previous antipsychotic treatment; psychotropic drugs with potential metabolic side effects and any other drug that might affect glucose or lipid homeostasis) was collected using an *ad hoc *schedule.

Assessments for the present analyses included fasting blood samples for glucose, cholesterol and triglycerides. The metabolic fasting sample was drawn between 8 and 10 a.m., after at least 8 hours' fasting, before medication administration. Plasma glucose, cholesterol and triglyceride levels were determined by enzymatic procedure applying the Roche/Hitachi Modular D-P automated chemistry analyzer and using the standard analytical system packs Glucose/God-pap, Cholesterol/CHOD cod-pap and Triglycerides/GPO-pap.

Weight, height and BMI were determined. Body mass index was computed as body weight (kg) divided by the square of height (m^2^). Height and weight for each patient in the study group were measured by CMHC nurses at the time of blood examination.

### Reference group

A reference group of adult men and women without any psychiatric history or antipsychotic treatment were randomly selected from outpatients suffering from hyperhydrosis and referred to the Department of Neurological Sciences in Bologna. Fasting plasma glucose, cholesterol and triglyceride levels were derived anonymously by chart review of these reference subjects. BMI was not available in the reference group, but the clinical charts reported if patients were overweight or obese or not. Thus it was known from the clinical charts that none of the patients in the reference group was overweight or obese.

### Statistical analyses

We used analysis of variance (ANOVA) to examine differences among the treatment groups and reference group in mean glycaemia and cholesterolaemia levels and to compare mean BMI levels among treatment groups. Since triglyceride levels were not normally distributed, median trigliceridaemia levels were compared among groups, using non parametrical analysis (Kruskal Wallis test).

At a second stage, by way of co-variants in ANCOVA analysis we included the sociodemographic variables which differed among groups. Alpha levels were considered significant when below 0.01 (Bonferroni corrected: 0.05/5 groups).

To compare the prevalence of hyperglycaemia, hypercholesterolaemia and hypertriglyceridaemia among groups we used chi square analysis and the Fisher exact text. We calculated the crude and adjusted Odds Ratio (OR) to evaluate the relative risk of hyperglycaemia, hypercholesterolaemia and hypertriglyceridaemia for SGA-treated patients compared to the reference group. At a later stage we calculated the crude and adjusted Odds Ratio (OR) of metabolic disorders for SGA-treated patients without schizophrenia, obesity or over-protracted SGA treatment, since these conditions were described as risk factors for metabolic disorders and could thus be confounding factors in our analysis [11-14].

Abnormal metabolic levels and BMI were defined in our study on the basis of the National Cholesterol Education Program (NCEP) [15] and World Health Organisation (WHO) [16] criteria as follows: 1) an abnormal BMI was defined as between 25 and 29.9 for overweight and equal to or greater than 30 for obesity; 2) an abnormal blood glucose level was defined as equal to or greater than 110 mg/dl (equal to or greater than 126 mg/dl for diabetes); 3) an abnormal blood cholesterol level was defined as equal to or greater than 200 mg/dl; 4) an abnormal blood triglyceride level was defined as equal to or greater than 150 mg/dl.

Finally we evaluated the Pearson bi-variate correlations between metabolic parameters and drug doses. For the whole analysis we used Statistica for Windows (Statsoft, Kernel Release 5.5).

## Results

### Demographic and clinical data

Table 1 shows the sociodemographic, clinical and treatment features of the study sample and of the reference group. Seventy-six patients were on maintenance treatment with SGA in the index period and all of them consented to receiving fasting blood glucose, cholesterol and triglyceride evaluations. This study sample was composed of 76 patients, 38 males and 38 females. The reference group was composed of 36 subjects, 19 females and 17 males. The psychiatric patients had a mean age of 44.6 ± 16, while the control group had a mean age of 37.3 ± 8.6 (*post hoc *treatment groups vs reference group: df 1,110, F 6.39, p = 0.0129). As expected, a large majority of the patients treated with SGA suffered from schizophrenia or other psychotic disorders.

**Table 1:** Demographic and clinical features

	**Reference** ** N = 36**	**Clozapine ** **N = 6**	**Risperidone** ** N = 20**	**Olanzapine** ** N = 31**	**Quetiapine** ** N = 19**
Male	17(47.2%)	4 (67.0%)	13 (65.0%)	10 (32.3%)	11 (57.9%)
Age (years)*	37.3 ± 8.6	37.2 ± 4.0	41.4 ± 15.1	47.3 ± 17.8	45.9 ± 15.4
Psychiatric Diagnosis					
Schizophrenia and other psychotic disorders		6 (100%)	15 (75%)	23 (74.2%)	10 (52.6%)
Affective disorders		0	5 (25%)	8 (25.8%)	9 (47.4%)

*df 4(107), F = 2.64, p = 0.038

### Treatment distribution

Table 2 reports the treatment characteristics of the study sample. Patients in the 4 treatment groups had a similar duration of SGA exposure: 77% were treated for less than one year (on average for six months), and the remaining 23% for more than one year. Treatment duration longer than one year showed a a differing distribution trend among treatment groups (risperidone n = 8, 42%; clozapine n = 2, 33%; olanzapine n = 5, 16%; quetiapine n = 2, 10.5%; chi-square = 6.8, df = 3, p = 0.077); *post hoc *analysis showed that significantly more patients treated with risperidone had a treatment duration longer than one year compared with other treatment groups (42% of patients treated with risperidone vs 16% of patients treated with other SGA, chi-sq = 5.5, df = 1, p = 0.019). Dosages were at the lower end of the normal range. The distribution of co-therapies with possible metabolic side effects did not differ among the four treatment groups. A significantly higher rate of patients with quetiapine were treated with SGA in the past and a lower proportion were drug naïve (table 2) compared with patients in other treatment groups. None of the patients in the treatment or reference groups was treated with hypoglycaemic or hypolipidaemic agents or other drugs that might affect glucose or lipid homeostasis.

**Table 2:** Treatments

	**Clozapine** ** N = 6**	**Risperidone** ** N = 20**	**Olanzapine** ** N = 31**	**Quetiapine** ** N = 19**
**SGA Medication**				
Dose (mg)	441.3 ± 142.9	2.7 ± 1.6	10.4 ± 5.8	330.3 ± 300.9
Duration (months)	12.7 ± 12.4	12.8 ± 10.7	9.8 ± 11.5	7.4 ± 3.9
**Other treatments with metabolic side effects^1^**	3 (50%)	3 (16%)	7 (23%)	5 (26%)
**Previous treatment**				
Naive*	0	10 (50%)	13 (42%)	2 (10%)
SGA**	3 (3%)	3(15%)	6(19%)	11 (58%)
FGA	3 (11%)	7 (35%)	12 (39%)	6 (32%)

*^1^Other treatments with metabolic side effects were first generation antipsychotics, carbamazepine, lithium, sodium valproate, mirtazapine*.

*chi-sq 11.7, df 3, p = 0.009

** chi-sq 11.5, df 3, p = 0.009

### Relationship between metabolic disorders and treatment

Table 3 shows the mean values of glycaemia, cholesterolaemia, triglyceridaemia and BMI. Differences among groups were evaluated including as co-variants age, previous treatment and treatment duration longer than one year, which differed among groups. A trend towards a significant difference in glucose mean levels was observed (p = 0.026) and *post hoc *analysis showed that the reference group had significantly lower glycaemia (df 1,107, F = 7.95, p = 0.006). Cholesterol mean levels were pathological in all groups and did not differ among groups. The triglyceride levels were non-normally distributed; thus we used non parametric analysis (median and Kruskal Wallis test). Median triglyceride levels were pathologic in the clozapine and quetiapine groups. We observed statistically significant differences in median triglyceridaemia among groups (p = 0.001); *post hoc *analysis highlighted that the reference group had significantly lower triglyceridaemia (df 1,112, H = 7.47, p = 0.006). We observed that mean BMI values were over the clinical threshold of overweight in all groups and over the clinical threshold of obesity in the clozapine group. BMI mean values differed among groups (p = 0.005); *post hoc *analysis revealed that patients with clozapine had a significantly higher BMI (df 1,69, F = 8.18, p = 0.006).

**Table 3:** Metabolic profile

	**Reference ** **N = 36**	**Clozapine ** **N = 6**	**Risperidone ** **N = 20**	**Olanzapine ** **N = 31**	**Quetiapine ** **N = 19**	**df**	**F(H)**	**P**
Glucose* (mg/dl)	85.8 ± 10.3	99.67 ± 14.5	95.2 ± 18.6	91.4 ± 14.1	97.2 ± 15.7	4(105)	2.9	*0.026*
Cholesterol* (mg/dl)	209.3 ± 39.1	223.0 ± 45.9	213.4 ± 41.2	215.8 ± 41.9	237.6 ± 46.9	4(105)	1.3	0.277
Triglycerides**(mg/dl)	75(60.122)	194.5 (108–205)	145 (108–207)	105 (84–143)	161 (92–244)	4(110)	18.9	**0.001**
**BMI**		35.3 ± 6.4	29.1 ± 5.9	26.7 ± 5.3	29.3 ± 5.2	3(69)	4.6	**0.005**

* *Means values adjusted fro age and previous treatments are given for glucose and cholesterol (Anova analysis);*

** *Unweighted median values, first and last quartile are given for triglycerides.(Kruskal Wallis: df 4(110), H = 18.9 p = 0.0008)*

Table 4 shows pathological cases in each group. Hyperglycaemia prevalence showed a trend towards significant difference among groups; *post hoc *analysis showed that as a whole patients treated with SGA had a trend towards higher prevalence of hyperglycaemia than the references group (chi-sq = 5.15, df = 1, p = 0.02; Fisher exact text p = 0.076). Of the patients with hyperglycaemia, none of the reference subjects and 4 SGA-treated patients (2 patients with olanzapine, 1 with risperidone and 1 with quetiapine) reached the glycaemia threshold of diabetes (126 mg/dl). The prevalence of hypercholesterolaemia did not differ among groups. The hypertriglyceridaemia rate differed significantly among groups; *post hoc *analysis showed that the prevalence of hypertriglyceridaemia was significantly greater in SGA-treated patients than in the reference group (chi-sq = 7.26, df = 1, p = 0.007; Fisher exact text p = 0.003). Treatments groups had a significantly different rate of obesity (p = 0.015); *post hoc *analysis showed that patients on clozapine had a significantly higher rate of obesity (clozapine n 5, 83%, others n 19, 27%; chi-sq 8.09, df = 2, p = 0.013; Fisher exact text p = 0.012). The rate of overweight did not differ among treatment groups (p = 0.2).

**Table 4:** Pathological cases

	**Reference ** **N = 36**	**Clozapine ** **N = 6**	**Risperidone ** **N = 20**	**Olanzapine ** **N = 31**	**Quetiapine ** **N = 19**	**chi-s**	**df**	**P**
**Hyperglycaemia **(110 ≤ glycaemia ≤ 125.9 mg/dl)	1 (3%)	2 (33%)	2 (10%)	2(7%)	4 (22%)	8.9	4	*0.06*
**Diabetes **(glycaemia ≥126 mg/dl)			1(5%)	2(6.5%)	1(5%)	2.6	4	0.6
**Hypercholesterolemia **(cholesterolemia ≥200 mg/dl)	22 (61%)	4 (67%)	10 (50%)	18 (58%)	16 (84%)	5.5	4	0.241
**Hypertriglyceridemia **(triglyceridemia ≥150 mg/dl)	5 (14%)	5 (83%)	9 (45%)	7 (23%)	10 (53%)	18.2	4	**0.001**
**Overweight **(25 ≤ BMI ≤ 29.9)		1 (17)%	7 (39%)	13 (42%)	11 (58%)	4.5	3	0.2
**Obesity **(BMI≥ 30)		5 (83%)	7 (39%)	6 (19%)	6 (32%)	15.8	3	**0.015**

The Odds Ratio (OR) of having a diagnosis of hyperglycaemia was 8 times significantly greater for patients treated with SGA than for the reference group (OR 7.90, 95%CI 1.00–62.70, p = 0.023); this risk, when adjusted for age, was still higher in SGA-treated patients (OR 6.12, 95%CI 0.75–49.73, p = 0.09). Moreover the odds of receiving a diagnosis of hypertriglyceridaemia was 4 times significantly higher for patients treated with SGA than for the reference group (OR 4.00, 95%CI 1.39–11.46, p = 0.007); this risk was not affected by adjusting for age (OR 4.00, 95%CI 1.39–11.46, p = 0.007). The OR of receiving a diagnosis of hypercholesterolaemia was not greater for SGA-treated patients than for the reference group (OR 1.09, 95%CI 0.48–2.49, p = 0.834). The higher risk of receiving a diagnosis of hyperglycaemia and hypertriglyceridaemia for SGA-treated patients compared to reference subjects decreased slightly when considering only the subgroup of patients without obesity or overweight and did not decrease for the subgroup of patients without a diagnosis of schizophrenia and treatment duration shorter than one year (data available on request).

Finally Pearson's correlation between SGA doses and study variables (BMI, glycaemia, triglyceridaemia and cholesterolaemia) was not significant (data available on request).

## Discussion

Our main findings are that all groups treated with SGA had a poorer metabolic profile – in particular, significantly higher mean glycaemia and triglyceridaemia than the reference group – while we did not find differences in mean glycaemia or mean triglyceridaemia levels among treatment groups. Patients treated with SGA have a 6 times higher risk of receiving a diagnosis of hyperglycaemia and a 4 times higher risk of receiving a diagnosis of hypertriglyceridaemia than the control group.

Despite the limitations of this small cross-sectional study, our findings are consistent with previous evidence in the literature, which suggests that patients treated with SGA may be at increased risk of hyperlipidaemia and hyperglycaemia than untreated people [15-22]. Although several studies suggest that such rates probably vary among specific antipsychotic drugs, our study did not find differences among the various treatment groups. However, we should consider that at present these differences are not well quantified. Using a general practice research database in the United Kingdom, Koro and colleagues [15,16] estimated the risk of hyperlipidaemia and diabetes associated with olanzapine to be 4.6 (95% CI 2.4–8.9) and 5.8 (95% CI 2.0–16.7) times the risk associated with no antipsychotic use. In this study supported by Bristol Myers, the risk associated with risperidone was much lower, at 1.1 (95% CI 0.6–2.1) and 2.2 (95% CI 0.9–5.2) times the risk associated with no antipsychotic use. In contrast, the study by Buse et al. [19], supported by Eli Lilly, using a prescription claims data base in the United States, found that the risk of diabetes associated with olanzapine (HR 3.0, 95% CI 2.6–3.5), clozapine (HR 3.3, 95% CI 1.4–8.0), as well as risperidone (HR 3.4, 95%CI 3.1–3.8) and quetiapine (HR 1.7, 95% CI 1.2–2.4), were higher than the risk associated with no antipsychotic use.

To our knowledge, among previous cross-sectional clinical studies which evaluated the mean glycaemia level in patients treated with SGA, only 2 carried out a comparison with untreated subjects [23,24]. Consistently with our results, these studies found that treated patients had a significantly higher fasting glycaemia level than control subjects; furthermore, again consistently with our study, such research did not find any significant differences among patients treated with different SGA (olanzapine, risperidone and clozapine) on the fasting glycaemia mean level. In turn, the Smith et al. study found that in patients treated with risperidone, olanzapine and clozapine mean fasting glucose was within the normal range and did not significantly differ across the drug treatment groups [25]. Among cross-sectional studies which evaluated fasting triglyceridaemia, only the Alméras et al. study, supported by Janssen [23], performed a comparison with untreated subjects. In contrast with our result, this study found differences among SGA and showed that olanzapine-treated patients, but not patients with risperidone, had a higher triglyceride level. Again, we did not observe a worse cholesterol profile in SGAs-treated patients compared to untreated subjects, consistently with studies suggesting that SGA have a relatively greater effect on blood triglycerides than on cholesterol levels [4-6]. Finally, we observed a higher BMI mean value in patients with clozapine than in those with 1^st ^line SGA. Although our result should be regarded carefully, since only 6 patients were treated with clozapine in our sample, this finding is in accordance with those of other clinical studies which showed the high impact of clozapine on body weight [11,26-35], as well as with the results of the Allison meta-analysis [36]. However, prospective clinical studies comparing different SGA indicate that weight gain induced by olanzapine is no less important than clozapine-induced weight gain [28,34,37,38], and greater than weight gain induced by other first line SGA (risperidone, quetiapine and ziprasidone), as recently shown by the CATIE trial [39].

In accordance with the data in the literature [11,26-28], we did not find any positive correlations between mean metabolic and BMI levels and SGA doses, which in our sample were towards the lower end of the normal range. Furthermore, in our study patients with shorter duration of SGA exposure had a similar risk of hyperglycaemia and hypertriglyceridaemia to that of the overall patient sample. These results are consistent with various evidence suggesting that SGA metabolic side-effects arise independently of treatment doses and duration [11,28,29]. Finally, our results showed that even SGA-treated patients without other known risk factors for metabolic disorders, such as obesity and schizophrenia, had a higher risk of hyperglycaemia and hypertriglyceridaemia than the control group. These results are consistent with several studies which observed SGA metabolic side-effects even in patients without any weight gain [30] and affected by mood disorders [31].

All in all, the evidence from published studies in the literature is not consistent as to the metabolic impact of different SGA. We found differing position statements even among expert Consensus Conferences: while the American Psychiatric Association distinguishes between SGA at higher and lower risk of causing metabolic side-effect [3], the Dublin Consensus Conference [8] and the Australian Consensus Conference [9] are more guarded and conclude that each SGA should be viewed with caution concerning its metabolic side-effects. Moreover, several studies claim that FGA as well, and severe mental disorders *per se*, increase the risk of metabolic disorders [12,13,19,22]. Since the risk of disturbances to both the glucose and the lipid metabolism and the risk of weight gain appear to be increased by all antipsychotic treatments and hard to differentiate among the various different SGA, according to the international consensus conferences [3,8,9] we recommend that monitoring of weight, fasting plasma glucose cholesterol and triglyceride levels should be performed in routine clinical practice with all antipsychotics.

### Limitations

The most important limitations of our study are due to the small sample size and the cross-sectional design. The small sample size may not have allowed us to clarify all possible differences among treatment groups. The cross-sectional study design did preclude determination of the temporal relationship between the prescription of SGA and the development of obesity and metabolic disorders. Thus the limitation due to the cross-sectional nature of this study makes it difficult to understand if these disorders are due to SGA itself or to other risk factors for diabetes mellitus and other metabolic disorders which are frequently observed in psychiatric patients, such as poor overall physical health, unhealthy life style, inadequate health care, familial and genetic vulnerability [40-42].

The lack of matching with the control group in terms of body composition, and the dearth of background variables available, limit the information that can be gleaned from such a comparison. We cannot assume that our control group is representative of the general population, since this sample was drawn from hyperhydrotic patients. Nevertheless, in our experience there is no evidence that patients suffering from idiopathic hyperhydrosis are at higher risk of diabetes or dyslipidaemia than the general population. In our sample SGA-treated patients were significantly older than the reference group. However, co-varying for age we found that age did not influence our results. This result is consistent with several studies reporting that age did not influence the risk of SGA metabolic disorders, which also occur in young patients [20,43]. Finally our results are limited by lack of information about some indexes of metabolic disorders, such as fat distribution and blood pressure, and other important clinical variables, such as the severity of psychiatric illness.

The relative level of risk associated with each type of SGA cannot be clearly defined by our cross-sectional study design. Only 25 (33%) patients were drug naïve before starting the studied treatment, while 23 patients (30%) had been treated in the past with different SGA and 28 (37%) with FGA. We should bear in mind that patients may have been switched from a previous antipsychotic following the development of obesity or metabolic disorders. Although we cannot rule out this potentially confounding variable, we have attempted to adjust for it by co-varying the effect of previous treatment. This did not change the results.

Finally we could not calculate the Relative Risk of developing metabolic disorders of SGA-treated patients as compared to FGA-treated patients, in view of the lack of FGA-treated patients in our sample.

## Conclusion

The health implications of long-term therapy with SGA that increase the risk of medical morbidity are of growing concern and may well be more dangerous than the extrapyramidal symptoms typically associated with older antipsychotic agents [6,40,41]. As a group, they are much more expensive than FGA, some of which are available as generic drugs. Unfortunately, at present independent studies clearly defining the metabolic risk factor profile associated with SGA use are thin on the ground and their findings are often inconsistent.

The rate of obesity and metabolic disorders observed in the current independent study was higher than the prevalence in our control group and in the general population as reported by epidemiological studies [44,45]. Furthermore this result is consistent with the prevalence found in extensive studies of psychiatric samples [36,46,47]. These findings implied *per se *that more attention should be paid to the metabolic condition of the psychiatric patients and that SGA treatment should be monitored routinely in clinical practice. In line with the International Consensus Conferences [3,8,9], we recommend that monitoring of weight, fasting plasma glucose levels, cholesterol and triglycerides be included in routine clinical practice with all antipsychotics. Considering the early emergency of metabolic side effects, this check-up should be done at the baseline and after the first month of treatment. After the initial period, evaluation of the metabolic side effects should be obtained every 6 months during the first year of treatment and then yearly. Moreover, as International Consensus Conferences have recommended [3,8,9], clinicians should consider any personal and family risk factors of obesity and metabolic disorders in patients treated with antipsychotic agents, such as a family history of diabetes. Particular efforts should be made to implement counselling about patients' lifestyles and behaviours, which could potentially limit the antipsychotic metabolic side-effects [48].

In a society in which cardiovascular disease continues to be a principal cause of morbidity and mortality, clinicians need to be aware of the metabolic risk factors when treating patients with mental disorders, and should weigh the pros and cons of an antipsychotic drug from each group, FGA and SGA, evaluating the relative benefits, risks and cost associated with specific choices.

## Competing interests

The author(s) declare that they have no competing interests.

## Authors' contributions

IT conceived of the study, designed the analytical plan, performed the statistical analysis, interpreted the results and drafted the manuscript. MC participated in study design, the analytical plan and interpretation of the results. BFG participated in study design, the analytical plan, statistical analysis, interpretation of the results and drafting the manuscript. DG participated in study design, the analytical plan, statistical analysis, interpretation of the results and drafting the manuscript. MM participated in designing the analytical plan, supervising the statistical analysis, interpreting the results, and reviewing the manuscript. AS supervised the statistical analysis, interpreted the results, and reviewed the manuscript. FP collected the reference group information and reviewed the literature on the topic of hyperhydrosis and metabolic disorders. MU reviewed the manuscript. DB participated in designing the analytical plan, interpreting the results and reviewing the manuscript. All authors read and approved the final manuscript.

## Pre-publication history

The pre-publication history for this paper can be accessed here:


